# Potential impacts of soil microbiota manipulation on secondary metabolites production in cannabis

**DOI:** 10.1186/s42238-021-00082-0

**Published:** 2021-07-03

**Authors:** Bulbul Ahmed, Mohamed Hijri

**Affiliations:** 1 Institut de Recherche en Biologie Végétale, Université de Montréal, 4101 Sherbrooke Est, Montréal, Québec H1X 2B2 Canada; 2 African Genome Center, Mohammed VI Polytechnic University (UM6P), Lot 660, Hay Moulay Rachid, 43150 Ben Guerir, Morocco

**Keywords:** *Cannabis*, Cannabinoid, Cannabidiol (CBD), Tetrahydrocannabinol (THC), Microbiota, Bioinoculants

## Abstract

**Background:**

Cannabis growing practices and particularly indoor cultivation conditions have a great influence on the production of cannabinoids. Plant-associated microbes may affect nutrient acquisition by the plant. However, beneficial microbes influencing cannabinoid biosynthesis remain largely unexplored and unexploited in cannabis production.

**Objective:**

To summarize study outcomes on bacterial and fungal communities associated with cannabis using high-throughput sequencing technologies and to uncover microbial interactions, species diversity, and microbial network connections that potentially influence secondary metabolite production in cannabis.

**Materials and method:**

A mini review was conducted including recent publications on cannabis and their associated microbiota and secondary metabolite production.

**Results:**

In this review, we provide an overview of the potential role of the soil microbiome in production of cannabinoids, and discussed that manipulation of cannabis-associated microbiome obtained through soil amendment interventions of diversified microbial communities sourced from natural forest soil could potentially help producers of cannabis to improve yields of cannabinoids and enhance the balance of cannabidiol (CBD) and tetrahydrocannabinol (THC) proportions.

**Conclusion:**

Cannabis is one of the oldest cultivated crops in history, grown for food, fiber, and drugs for thousands of years. Extension of genetic variation in cannabis has developed into wide-ranging varieties with various complementary phenotypes and secondary metabolites. For medical or pharmaceutical purposes, the ratio of CBD to THC is key. Therefore, studying soil microbiota associated with cannabis and its potential impact on secondary metabolites production could be useful when selecting microorganisms as bioinoculant agents for enhanced organic cannabinoid production.

## Introduction

The *Cannabis* genus is comprised of two major groups of accessions: the *indica* gene pool and the *sativa* gene pool, which are closely related subspecies of *Cannabis sativa* L. — although between these two subspecies, diverse cultivars have been domesticated throughout the hybridization process (Small and Cronquist, 1976; Emboden, 1981; Hillig, 2005). Regardless of evolutionary relationships, *Cannabis* is largely cultivated for medicinal and recreational use, and this has led to further categorization with regard to its relative cannabinoid concentrations, which broadly vary between the male and female plants (Small and Cronquist, 1976; Emboden, 1981; Hillig, 2005). Over time, cultivation and breeding of *Cannabis* plants led to the expansion of genetic variations, resulting in a range of cultivars with contrasting phenotypes, traits and secondary metabolite properties (Li, 1973; Clarke and Merlin, 2017; Saloner and Bernstein, 2020; Danziger and Bernstein, 2021a, b; Shiponi and Bernstein, 2021). Despite technological advances in *Cannabis* breeding, the proportion of cannabinoids — or more specifically, of cannabidiol (CBD) and tetrahydrocannabinol (THC) — fluctuate greatly depending on various factors, including the sex of the parents (male or female), genotypes, cultivation practices, and biotic or abiotic stresses (Backer, et al., 2019; Saloner and Bernstein, 2020; Danziger and Bernstein, 2021a, b). As a source of biotic stress, plant chemical compounds formed by signaling molecules from living organisms, as well as nutrient deficiency, water, and salt as an abiotic stressor may influence plant enzymatic pathways, altering the content of secondary metabolites (Pate, 1994; Gorelick and Bernstein, 2014, 2017; André, et al., 2020). That said, stress responses impact secondary metabolites such as alkaloids (Balsevich, et al., 1986; Facchini, 2001), terpenes (Trapp and Croteau, 2001; Pichersky and Raguso, 2018), and phenylpropanoids (Dixon and Paiva, 1995; Sharma, et al., 2019; Dong and Lin, 2021). *Cannabis* plants have attracted much attention for medical uses due to the importance of secondary metabolites, for which demand increased in the last decade following the discovery of the main compound present in *Cannabis sativa* — cannabinoids. Cannabinoids, a class of compounds specific to cannabis, are responsible for the vast majority of its medicinal activity (Gaoni and Mechoulam, 1964; Gorelick and Bernstein, 2017; Freeman, et al., 2019; Nelson, et al., 2020; LaVigne, et al., 2021). Here, we will simplify the potential of microbes for cannabinoid production, and how they could be manipulated for the stabilization of biosynthesis of different cannabinoids.

### Factors influencing the production of cannabinoids

Cultivation methods and different abiotic and biotic factors are important considerations for cannabinoid biosynthesis. Variation in secondary metabolites of plant material is influenced by abiotic factors, for example, light, temperature, humidity, water availability, and nutrients (Krejci, 1970; Haney and Kutscheid, 1973; Bazzaz, et al., 1975; Coffman and Gentner, 1977; Valle, 1978; Lydon, et al., 1987; Pate, 1994; Bócsa, et al., 1997; Kakani, et al., 2003; Chandra, et al., 2011; Marti, et al., 2014; Magagnini, et al., 2018; Bernstein, et al., 2019; Landi, et al., 2019). As is the case for other plants, the growth and metabolism of cannabis are heavily affected by light spectra (Eaves, et al., 2020; Danziger and Bernstein, 2021a, b), with blue and red light having a significant impact on cannabinoid metabolism (Danziger and Bernstein, 2021a, b). Secondary metabolites including phenols, terpenes, flavonoids and anthocyanins are stimulated by UV radiation (Kakani, et al., 2003; Caldwell, et al., 2007; Backer, et al., 2019; Eichhorn Bilodeau, et al., 2019). UV-B radiation has been shown to increase THC concentrations (Lydon, et al., 1987) and UV-C radiation has been demonstrated to enhance the biosynthesis of cinnamic acids in cannabis (Marti, et al., 2014). Non-nitrogenous shikimic acid-dependent metabolites are more readily synthesized in the absence nutrients (Fluck, 1963; Waterman and Mole, 1989; Berenbaum, 1995, Nascimento NC and Fett-Neto, 2010). While the effect of nutrients on cannabinoid production is still not clear, soil conditions (Cooper, 2021); mineral and biostimulant substance amendments, for example nitrogen, iron, calcium and magnesium (Haney and Kutscheid, 1973; Kaneshima, et al., 1973; Latta and Eaton, 1975; Gorelick and Bernstein, 2017); and humic acids and NPK (Bernstein, et al., 2019) have been suggested as factors influencing THC content. NPK fertilizers have been shown to increase cannabigerol (CBG) concentrations by 71% in flowers and to decrease cannabinol (CBN) concentrations by 38% and 36% in flowers and inflorescence leaves, respectively. However, humic acids were shown to minimize the normal spatial heterogeneity of all cannabinoids analyzed (Bernstein, et al., 2019). Poor soil (Krejci, 1970), inadequate potassium, and moisture were found to increase THC production in hemp plants (Haney and Kutscheid, 1973). The effect of temperature on the production of cannabinoid is even more complicated because it is strain dependent (BRAUT, 1980) and may have both positive (Boucher, et al., 1974) and negative (Bazzaz, et al., 1975) effects in production of cannabinoids. Photomorphogenic behavior largely influences synthesization of cannabinoids in the glandular trichomes (Potter, 2009; Darko, et al., 2014; Eichhorn Bilodeau, et al., 2019).

Cannabinoids are unique secondary metabolites to *Cannabis* and are produced by trichomes of the plant. Although 61 true biosynthetic cannabinoids exist, *Cannabis* is generally cultivated for its CBD and THC, for both medicinal and recreational purposes. Previous studies have documented that the cannabinoid content in plants varies greatly depending on climatic conditions, plant genotypes, and cultivation practices that influence cannabinoid biosynthesis pathways (Small and Cronquist, 1976; Beutler and Marderosian, 1978). For example, high concentrations of THC in *Cannabis* plants were reported in cultivars originating from India, Nepal, Eastern Asia, and Southern Africa, while high concentrations of CBD were found in Northeast Asian cultivars (Fetterman and Turner, 1972; Small and Beckstead, 1973a, b; Small and Beckstead, 1973a, b; Turner and Hadley, 1973a, b; Turner and Hadley, 1973a, b; Baker, et al., 1980). However, as *Cannabis* is widely grown in indoor conditions with diverse growing substrates, artificial light, and temperature control, these factors are not sufficient to stabilize ratios of THC and CBD in the plants, which brings another level of complexity to cannabinoid production and standardization. Many possibilities have been explored by *Cannabis* growers in the effort to find a way to maintain the yield (Backer, et al., 2019) and safeguard production against pathogens (Taghinasab and Jabaji, 2020; Vujanovic, et al., 2020); however, scientific research for stabilization or balanced proportions of THC and CBD has not been reported. Among these solutions, biostimulant substances, beneficial microbes belonging to plant growth-promoting rhizobacteria (PGPR), and arbuscular mycorrhizal fungi have been proposed. Owing to previous legal limitations on cannabis cultivation, there is a lack of evidence on the use of microbes in cannabis production. Apart from that, microbes play an important role in the biosynthesis of cannabinoids and a deeper understanding of the relationship between microbes and production of cannabinoids is critical.

### Why should we consider microbial interactions for cannabinoid production?

Advances made in high-throughput sequencing technologies offer possibilities for manipulating soil and plant microbiota to enhance crop yield and sustain agroecosystems (Ercolini, 2013; Lucaciu, et al., 2019; Fadiji and Babalola, 2020). Microbiota manipulation refers here to human intervention to alter the taxonomic composition and abundance of microbial communities associated with *Cannabis* plants. Bacteria and fungi are the two most important microbiota groups that closely or loosely interact with plants in a beneficial or adverse manner. Plants and their associated plethora of microbes nurture multifactorial interactive relationships where specific microorganisms including bacteria and fungi can stimulate the biosynthetic and signaling pathways of the host plants for the production of pharmaceutically or agronomically important metabolic compounds (Scherling, et al., 2009, van de Mortel, et al., 2012, Huang, et al., 2014, Ryffel, et al., 2016, Pascale, et al., 2019). Recent literature has shown that root-associated microbes stimulate the systematically induced root exudation of metabolites (SIREM) process and affect levels of root transcriptomes and metabolomes (Korenblum, et al., 2020). Endophytic bacteria and fungi can influence the metabolic machinery for producing a specific medicinal compound. For example, the pharmaceutically essential terpenoid indole type alkaloids vindoline, serpentine, and ajmalicine showed a substantial increase when Madagascar periwinkle (*Catharanthus roseus* L.) plants were inoculated with the endophytic bacteria *Staphylococcus sciuri* and *Micrococcus* sp. (Etalo, et al., 2018). Recent analysis (Taghinasab and Jabaji, 2020) addressed the use of exogenous inducers such as phytohormones abscisic acid (ABA), gibberellins (GA), and ethylene (ET) on the possible recovery of secondary metabolites in cannabis. Few bacterial (*Pseudonomas fulva* BTC8-1, *P*. *orientalis* BTG8-5, and *Panibacillus sp*. MOSEL-w13) and fungal endophytes (*Penicillium copticola* L3, *Paecilomyces lilacinus* A3, and *Alternaria niger* 2) in cannabis have demonstrated their potential biocontrol effects against *Trichothecium roseum*, *Botrytis cineria*, *Fusarium solani*, *Curvularia lunata*, *Aspergillus niger*, and *Fusarium oxysporum* (Kusari, et al., 2012; Gautam, et al., 2013; Qadri, et al., 2013; Afzal, et al., 2015; Scott, et al., 2018). It has been shown that transferring PGPR from one crop to another plant species may stimulate yield and biocontrol effects (Smith, et al., 2015; Backer, et al., 2019), and the collective role of endophytes with the exogenous application of inducers in cannabis could stimulate improvement of THC and CBD content, though its correlation and mechanism have not yet been fully revealed (Taghinasab and Jabaji, 2020). These authors did not discuss how the entire microbiome (not only the endophytes) associated with cannabis root could be effectively used in manipulation of the cannabinoid profile. PGPR has attracted scientists’ attention for its potential to increase the quality and quantity of production of desired cannabinoids; however, in most cases, scientists have focused on well-known PGPR genera, for example, *Pseudomonas* and *Bacillus* for cannabinoid yield and disease control effects demonstrated in other crops (Lyu, et al., 2019). Microbiota are genotype-specific for a variety of beneficial functions, such as nutrient acquisition, stress response, pathogen tolerance, and secondary metabolite biosynthesis (Liu, et al., 2019; Brown, et al., 2020). As a result, complex signal coordination between the host and associated microbes is evident in particular plant–microbe interactions, contributing to overall understanding of plant-specific microbial inputs. Different microbes can colonize various areas of the root, increasing the total root biomass and nutrient acquisition capacity. Some microbial strains protect plants against pathogen attacks, while others improve the resilience and recovery of plants subjected to stress. Advances made in high-throughput sequencing and bioinformatics have made it possible to search for microbial genes present in a given environmental sample for novel functions that can influence the production of bioactive compounds (Fernández-Arrojo, et al., 2010). Beneficial microbes recruited by plant genotypes can be discovered using diversified microbial communities derived from the natural environment. For example, natural microbial amendments from undisturbed and old growth maple forest organic soil showed an increased phosphorus acquisition in soybean plants (unpublished data). Since the interplay between the microbes and plant stimulates various biochemical pathways, leading to the production of secondary metabolites, as a result, decoding the community profile of cannabis-associated microbes would produce a large list of microbes to choose from and its microbiota would unravel the complexity of stabilizing cannabinoid production.

### Current knowledge in microbiota research related to cannabinoids

The first draft genome sequencing of *Cannabis* solved one of the utmost ambiguities — that although they contain divergent pharmaceutical compounds, marijuana and hemp are derived from a single species: *Cannabis sativa L*. In addition, the genomic map provides clues to the scientific community for accelerating breeding programs to develop new cultivars with improved properties (van Bakel, et al., 2011). The first report on the *Cannabis* plant microbiome highlighted cultivar-specificity and soil determinants of the microbiome for five *Cannabis* cultivars — Bookoo Kush, Burmese, Maui Wowie, White Widow, and Sour Diesel — and reported a core bacterial community composed of *Pseudomonas*, *Cellvibrio*, *Oxalobacteraceae*, *Xanthomonadaceae*, *Actinomycetales*, and *Sphingobacteriales* (Winston et al., 2014). This study included the biochemical correlations with bacterial communities, highlighting that the concentration and composition of CBD were correlated with the structure of bacterial communities residing inside the root system, whereas THC concentrations were correlated with the soil’s edaphic factors. Another study on three *Cannabis sativa* L. (industrial hemp) cultivars grown in Quebec (Anka, CRS-1, and Yvonne) reported 18 bacterial and 13 fungal endophytic isolates, of which three bacterial genera of *Pseudomonas*, *Pantoea*, and *Bacillus* and three fungal genera of *Aureobasidium*, *Alternaria*, and *Cochliobolus* were found to be widely distributed in the above-ground tissues (Scott, et al., 2018). Further experiments are needed to validate the effects of these isolates on *Cannabis* growth and secondary metabolite production. Of the microbial inoculants engineered for *Cannabis* production, Mammoth P™ is an example of microbial biostimulants used to improve bud growth, yield, and plant biotic stress (Conant, et al., 2017). The authors reported that using Mammoth P™ in *Cannabis sativa* resulted in a 16.5% increase in plant aerial biomass, but no connection to cannabinoid synthesis was found.

The first report on spatiotemporal and cultivar-dependent divergences in indoor commercial settings showed variations in the bacterial and fungal microbiome of *C*. *sativa*. The study included three cultivars — CBD Yummy, CBD Shark, and Hash — and was carried out in strict indoor commercial settings (Comeau, et al., 2020). The experiment was conducted without the use of microbial inoculants. The study investigated spontaneous microbes that established themselves during the growth of *Cannabis* plants. Nonetheless, they found that microbial communities evolved over time and differed between *Cannabis* strains. *Penicillium*, *Aspergillus*, *Zopfiella*, and *Fusarium* genera of Ascomycota and Basidiomycota were recognized as the dominant fungi while Burkholderiaceae and Rhizobiaceae of the phylum Proteobacteria, and Streptomycetaceae and Norcardiodaceae of the phylum Actinobacteria were the dominant bacteria. The metabolic profiling linking microbes associated with the rhizosphere were not quantified in the study. The metabolic pathway was predicted based on bacterial abundance linked to glucose, pentose, lipid, and amino acid metabolism, but this was not verified (Comeau, et al., 2020).

Plant microbiomes are dynamic and can change in response to external factors. The first study of PGPR application showed positive impact on CBD and THC content in *C*. *sativa* “Finola” (Pagnani, et al., 2018); however, microbes modulating plant metabolites in a number of other crops can be applied to cannabis. For example, a consortium containing *Bacillus* sp., *Streptomyces* sp., *Pseudomonas* sp., *Azospirillum* sp., and an arbuscular mycorrhiza fungus *Glomus* sp. has been reported to affect the composition of secondary metabolites in maize (Walker, et al., 2011, 2012; Couillerot, et al., 2013). *Pseudomonas aeruginosa* PJHU15, *Bacillus subtilis* BHHU100, and *Trichoderma harzianum* TNHU27 increased phenol levels in pea plants (Jain, et al., 2015). Root-secreted secondary metabolites play a significant role in plant-soil microbiome interactions (Sasse, et al., 2018). Some microbes have the potential to adjust individual output by influencing the local environment (Köberl, et al.,^ 2013^, Hu, et al., 2018, Huang, et al., 2018). Secondary metabolites in maize, such as benzoxazinoids, have been shown to attract Chloroflexi bacteria and manipulate the assembly of maize microbiomes, which enhanced the resilience of maize plants against stress (Hu, et al., 2018). *Methylobacterium* sp. has been found to play a role in modulating the production of flavor-related phytometabolites (Brader, et al., 2014). *Trichoderma harzianum* has been shown to increase the root system (Harman, 2011; Guzmán-Guzmán, et al., 2019; Vicente, et al., 2020), biomass accumulation, and water content (Oljira, et al., 2020) in a variety of crops including wheat, soybean, and cucumber. In the same way, *T*. *harzianum* increased CBD content as well as biomass in hemp (Kakabouki, et al., 2021).

### Determining core microbiota could enhance and sustain cannabinoids

In this context, the core microbiota is defined as a microbial community common and essential to all healthy *Cannabis* plants. This core microbiota is anticipated to give indispensable indicators of crucial soil processes, of links between microbiota and their functional attributes (Delgado-Baquerizo, et al., 2018), and of soil microbial communities (Zamioudis and Pieterse, 2011; Lebeis, 2014). Plant genotype plays a key role in shaping the microbial communities of the rhizosphere (Marques, et al., 2014; Sapkota, et al., 2015). Plant root bound microbes are so crucial for plant health that they are often referred to as the second genome of the plant (Berendsen, et al., 2012). As a result, selecting microbes that enhance nutrient security, plant health, and chemical compound biosynthesis for a particular genotype is critical. Despite being linked to below-ground nutrient cycling, genotype plays a vital ecological role in maintaining complex interactions between microbial taxa. However, due to competition with native soil microbial communities or impaired colonization performance, the introduction of unspecified microbes may be ineffective or have an antagonistic effect (Qin, et al., 2016). The core microbiota could be a baseline for selecting beneficial microbes, whose communities could then be manipulated to enhance the desired functions and services of plant hosts such as biochemical compound production. To do so, we must prioritize studies of the composition of the cannabis microbial community and the factors that influence it at different stages of development. Microbial populations are diverse and are influenced by growth stages and cultivation conditions. Many studies examine a variety of root-associated microbial communities — for example, mycorrhizal fungi and their associated microbes — that promote plant growth in an agricultural context (Ismail and Hijri, 2012; Hijri, 2016; Zarik, et al., 2016) or the microbial community in contaminated environments (Hassan, et al., 2013; Bell, et al., 2014; Bourdel, et al., 2016; Iffis, et al., 2017; Dagher, et al., 2019, 2020). Such studies could be adapted and applied to marijuana and industrial hemp so as to decipher the underlying core microbiota.

**Fig. 1:**
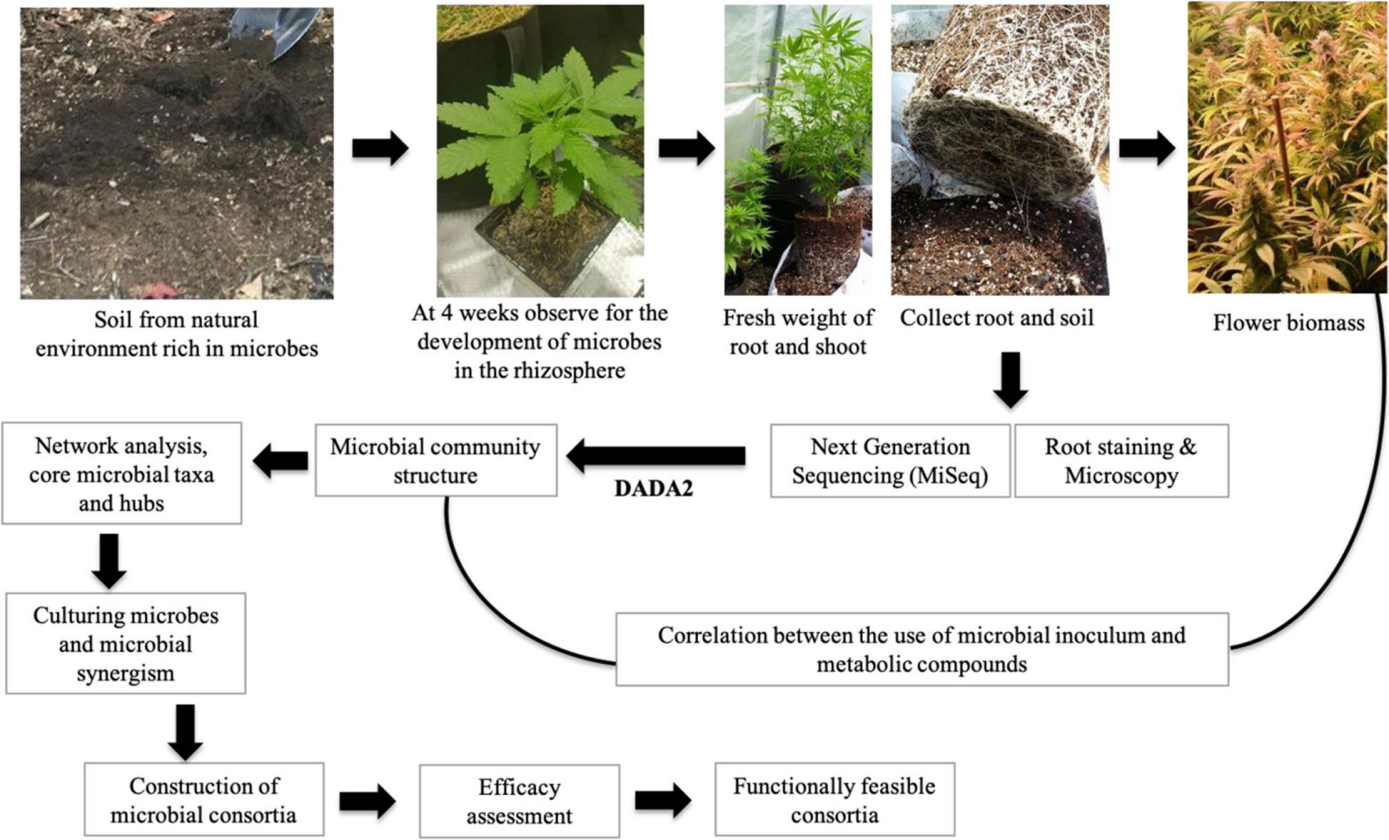
This illustration shows the technical workflow for the development of microbial consortia for *Cannabis* (hemp and marijuana). The source is a microbial suspension from a natural environment rich in microbes. A root colonization study at 4 weeks allows assessment of whether microbes have been established. Next-generation sequencing and the DADA2 pipeline in R can determine community composition, and core microbes can be isolated from cannabis roots and rhizospheric soils. Network analysis will provide insight into the core microbial taxa and hub microbes. Later, the core microbes can be cultured in different microbial growth mediums and microbial synergism can be evaluated. Then, different microbial consortia can be considered for efficacy assessment, and functionally feasible consortia considered for utilization in the cultivation of marijuana and hemp

Many recent studies have shown the potential of manipulation of soil microbial communities to enhance yields and sustain agroecosystems for different crops, such as corn, wheat and soybeans (Renaut, et al., 2020); blueberries (Morvan, et al., 2020); and canola (Floc’h, et al., 2020). A study demonstrated that a natural microbial suspension prepared from an undisturbed old maple forest and used as a soil amendment increased soybean biomass and phosphorus acquisition by hydrolyzing phytate (unpublished data). This suggests that microbe-rich natural soil could be applied as a soil amendment to manipulate cannabinoid production in cannabis. Here, we present a technical workflow for decoding the microbial community structure of *Cannabis* and using it to identify core microbiota, which can then be tested and validated for cannabinoid production. Members of the core microbiota should be tested for compatibility, synergy, plant growth-promoting activities, and positive correlation with secondary metabolite production. We propose that *Cannabis* plants recruit their specific core microbiota when they are inoculated with a microbial suspension prepared from naturally microbial-rich environments such as forest soils. Amplicon sequencing targeting the bacterial 16S rRNA gene, the fungal ITS gene, and the fungal 18S rRNA gene, coupled with whole metagenome and/or metatranscriptome using high-throughput sequencing (e.g., Illumina platforms), will allow us to decipher the microbial communities and their spatiotemporal changes in different *Cannabis* cultivars. Ecology of soil microbial network analyses within community (Kurtz, et al., 2015) and interkingdom interaction (Hartman, et al., 2018; Floc’h, et al., 2020) correlation based on the occurrence of microbial species will assist us in finding hub microbial taxa across cultivars. These hub taxa will be the candidates for identifying core microbes influencing plant growth and cannabinoid biosynthesis (Fig. 1), while evidence of the hub microbial species will serve as a first step toward screening microbes exclusively for cannabinoid stabilization.

## Conclusions

*Cannabis* has been banned by various countries around the globe over many centuries, limiting research and development of its cultivation. *Cannabis* cultivation is highly variable on a global scale, but cultivation practices can greatly influence yield and cannabinoid quality even in the same cultivar. Mounting concerns regarding stable and sustained cannabinoid production have recently drawn the focused attention of scientists. Our main purpose here is to speculate on the most sustainable way to minimize the imbalance of cannabinoid production in *Cannabis*. Therefore, contingent upon high-throughput research, we propose that studies to decode microbial communities and their interactions with *Cannabis* plants would be a promising way to formulate bioinoculants for improvement of cannabis quality in sustainable agricultural practices. Although beneficial microbes for biomass improvement are available for other plant species, the practice of microbe-based organic farming for cannabis cultivation is still in its infancy. We propose that the identification of core microbiota and their correspondence with secondary metabolites production through metagenomics in combination with metabolomics will offer new leads for exploring the underlying mechanisms of *Cannabis* cultivation for improved, sustainable, and stable production of cannabinoids. Conclusively, deeper understanding of microbiota-biochemical talk may rationalize our current gaps in knowledge regarding correlating microbial mechanisms for stabilization of cannabinoid biosynthesis.

## Data Availability

Not applicable.

## Abbreviations

CBD: Cannabidiol
CBG: Cannabigerol
CBN: Cannabinol
THC: Tetrahydrocannabinol
PGPR: Plant growth-promoting rhizobacteria
SIREM: Systematically induced root exudation of metabolites
ABA: Abscisic acid
GA: Gibberellins
ET: Ethylene
